# KDM5B focuses H3K4 methylation near promoters and enhancers during embryonic stem cell self-renewal and differentiation

**DOI:** 10.1186/gb-2014-15-2-r32

**Published:** 2014-02-04

**Authors:** Benjamin L Kidder, Gangqing Hu, Keji Zhao

**Affiliations:** 1 Systems Biology Center, National Heart, Lung and Blood Institute, National Institutes of Health, Bethesda, MD 20892, USA

## Abstract

**Background:**

Pluripotency of embryonic stem (ES) cells is controlled in part by chromatin-modifying factors that regulate histone H3 lysine 4 (H3K4) methylation. However, it remains unclear how H3K4 demethylation contributes to ES cell function.

**Results:**

Here, we show that KDM5B, which demethylates lysine 4 of histone H3, co-localizes with H3K4me3 near promoters and enhancers of active genes in ES cells; its depletion leads to spreading of H3K4 methylation into gene bodies and enhancer shores, indicating that KDM5B functions to focus H3K4 methylation at promoters and enhancers. Spreading of H3K4 methylation to gene bodies and enhancer shores is linked to defects in gene expression programs and enhancer activity, respectively, during self-renewal and differentiation of KDM5B-depleted ES cells. KDM5B critically regulates H3K4 methylation at bivalent genes during differentiation in the absence of LIF or Oct4. We also show that KDM5B and LSD1, another H3K4 demethylase, co-regulate H3K4 methylation at active promoters but they retain distinct roles in demethylating gene body regions and bivalent genes.

**Conclusions:**

Our results provide global and functional insight into the role of KDM5B in regulating H3K4 methylation marks near promoters, gene bodies, and enhancers in ES cells and during differentiation.

## Background

Embryonic stem (ES) cells express a unique network of transcription factors (TFs) and epigenetic modifying enzymes that allow for indefinite self-renewal or differentiation into the many cell types that exist in mammals. The precise control of gene expression by epigenetic regulation of transcription is important for the maintenance of ES cell self-renewal or differentiation. Cell fate decisions of ES cells are controlled in part by external signals that regulate the expression of TFs and epigenetic modifiers, which in turn modify the underlying chromatin structure in a way that is conducive or repressive for transcription. ES cells express networks of TFs, such as Oct4, Sox2, Nanog, and Tbx3 that regulate self-renewal and differentiation by occupying promoters and enhancers to activate gene expression of ES cell-enriched genes and to repress developmental genes [1-3]. Perturbation of these core TFs results in the collapse of the self-renewal network, which has been suggested to promote differentiation [4]. While the roles of many TFs in ES cell self-renewal have been evaluated, the functions of epigenetic modifiers in ES cell pluripotency have not been fully explored [5-7]. Posttranslational modification of histone tails impacts the activity of epigenetic modifiers and the transcriptional state (active or inactive) of the underlying chromatin, which is important for controlling expression of networks of genes that promote self-renewal or differentiation.

The trithorax group (*trxG*) complex controls methylation of lysine 4 of histone H3 (H3K4me), which is associated with active genes [8]. H3K4me3 is predominantly localized to transcriptional start sites (TSSs) of highly expressed genes [9-12] where it plays a role in RNA polymerase II (RNAPII) binding and target gene activation [13-15]. In ES cells, methylation of H3K4me3 is facilitated by the mammalian homolog of *trxG*, including members of the Set/MLL histone methyltransferase (HMT) family. Wdr5 is a core subunit of the MLL complex that is essential for development [16], ES cell self-renewal [5], and induced pluripotent stem (iPS) cell reprogramming [5]. Demethylation of H3K4me3 is administered by the lysine demethylase 5 (KDM5/JARID1) family of jumonji (J) C containing protein complexes [17]. KDM5 demethylases remove H3K4 methylation, and are generally thought to be transcriptional repressors [18-20]. KDM5B (JARID1B) catalyzes the demethylation of tri-, di- and mono-methylation states of H3K4, as opposed to LSD1 (KDM1), which demethylates the di- and mono-methylation forms of H3K4, and H3K9me2 [21].

While multiple KDM5 family demethylases recognize similar histone modifications, each enzyme serves a unique function by associating with different complexes and by its mutually exclusive expression pattern throughout development. For example, KDM5C (SMCX) associates with HDAC1-2, G9a, and REST [22], whereas KDM5D (SMCY) forms a complex with Ring6a (polycomb-like) [18]. KDM5B is mainly expressed during development and in adult tissues such as testis, thymus, brain, spleen, and eye [23,24]. It is also highly expressed in stem cells, including ES cells [25-27], neural progenitors [25,26], trophoblast stem cells [28], and blood lineages [29].

Previous studies have shown that KDM5B is important for normal embryonic development [30,31], and two studies have described contrasting roles for KDM5B as essential [27] or dispensable [26] for ES cell self-renewal and differentiation. Therefore, to clarify the role of KDM5B in regulating ES cell pluripotency, we thoroughly studied the function of KDM5B-depleted ES cells and found that these cells have slightly reduced self-renewal and impaired differentiation [32]. Our results also showed that KDM5B is important for silencing pluripotency genes during differentiation, where KDM5B-depleted ES cells displayed extended self-renewal under differentiation conditions, and an inability to fully deactivate pluripotency regulators [32]. We also observed underexpression of bivalent genes during differentiation in the absence of KDM5B, thus implicating a role for KDM5B in regulating gene expression of bivalent genes during differentiation [32]. These findings suggest that KDM5B plays an important role in regulating ES cell pluripotency, although the precise role of KDM5B in regulating chromatin during this process is not understood.

Therefore, to clarify how the regulation of H3K4 methylation by the histone demethylase KDM5B contributes to ES cell function, we investigated the genome-wide occupancy of KDM5B in ES cells. Our data indicate that KDM5B co-localizes with H3K4 methylation marks at enhancers and promoters of active genes, functions to focus H3K4 methylation near these regulatory regions by preventing them from spreading to gene bodies and enhancer shores, and is critical for enhancer activity in ES cells. Additionally, we show that KDM5B regulates H3K4 methylation at bivalent (H3K4me3/H3K27me3) developmental genes during differentiation. We also report that KDM5B and LSD1 co-regulate H3K4 methylation at active promoter regions in ES cells, but their functions remain distinct at bivalent developmental genes and within gene body regions. Overall, our findings describe a novel mechanism by which KDM5B regulates ES cell pluripotency by modulating chromatin in a way that is conducive for differentiation.

## Results

### KDM5B co-localizes with H3K4me3 and pluripotency-related transcription factors at active genes in ES cells

**Figure 1:**
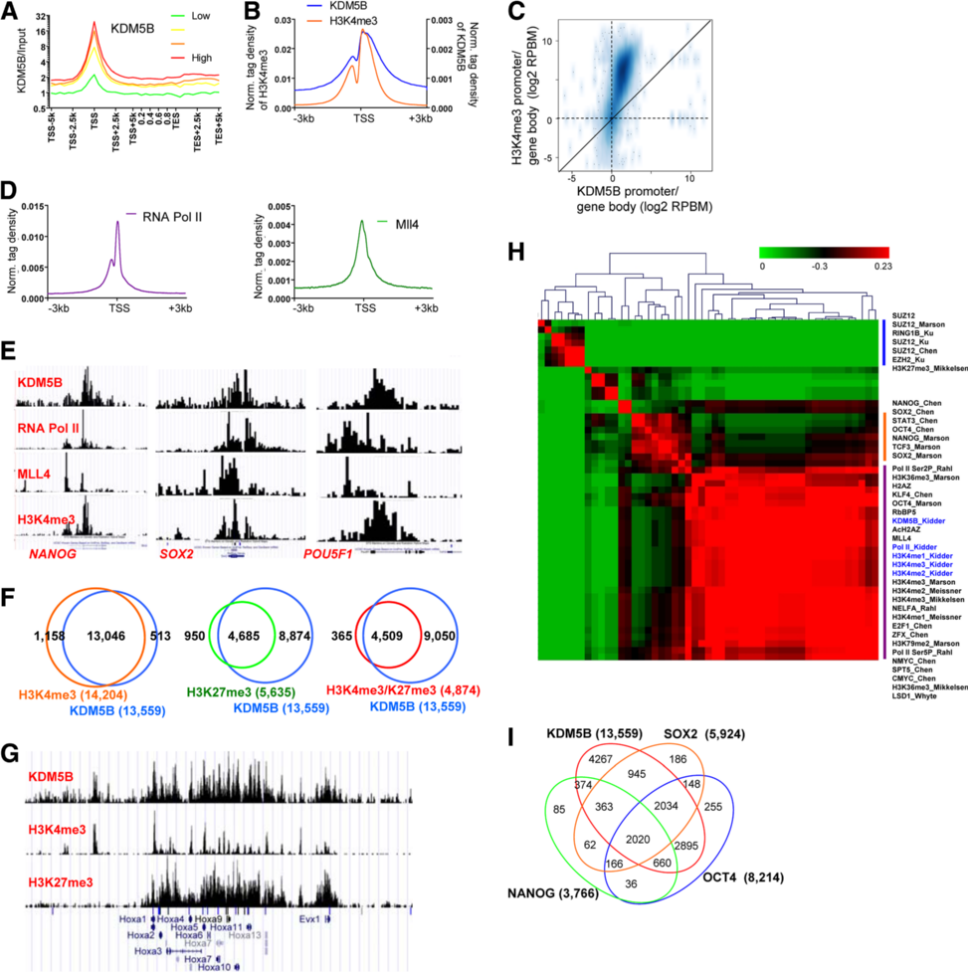
**KDM5B occupies active genes, pluripotency regulators, and bivalent genes in ES cells.** KDM5B is associated with transcriptional start sites (TSSs) and gene body regions of highly expressed genes in ES cells. **(A)** ChIP-Seq tag density of KDM5B binding at TSS normalized by input (log2 scale) of all refseq genes sorted into quartiles based on their mRNA expression level in ES cells. **(B)** ChIP-Seq tag densities of KDM5B and H3K4me3 around TSSs in ES cells. KDM5B binding profiles are similar to H3K4me3 marks near TSS regions, while KDM5B occupancy is enriched more in gene body regions relative to H3K4me3. **(C)** Scatter plot of the ratio of relative tag densities of KDM5B and H3K4me3 in promoter versus gene body regions. **(D)** RNA polymerase II and MLL4 are also enriched at TSS regions. **(E)** KDM5B occupies promoters of pluripotency-related genes in ES cells (Pou5f1/Oct4, Sox2, and Nanog). ChIP-Seq binding profiles of KDM5B, H3K4me3, RNA polymerase II, and Mll4 at core pluripotency genes. **(F)** Venn diagrams showing the co-occupancy of KDM5B and H3K4me3 (left panel), H3K27me3 (middle panel), and both modifications (right panel) at promoter regions. **(G)** Example of KDM5B binding at promoters marked with H3K4me3 and H3K27me3 (for example, HoxA cluster). **(H)** Correlation matrix of KDM5B binding with an assortment of TFs and epigenetic modifiers that are highly expressed in ES cells. Hierarchical clustering heat map generated by evaluating pair-wise affinities at promoters between ChIP-Seq datasets generated from this study (KDM5B, H3K4me3, RNAPII) and from published datasets [3,35-39]. AutoSOME [40] was used to generate pair-wise affinity values. **(I)** Venn diagrams showing co-occupancy of KDM5B, OCT4, SOX2, and NANOG at promoter regions.

We investigated the genome-wide distribution of KDM5B binding sites in ES cells using ChIP-Seq. Our results revealed that KDM5B binds to promoters, enhancers, and gene body regions of active genes as assessed by average profiles of KDM5B binding at transcriptional start and end sites (TSS-TES) of active genes (Figure 1A, red line, highest 25% expressed) and inactive genes (Figure 1A, green line, lowest 25% expressed). A comparison of average KDM5B binding and H3K4me3 marks around TSSs showed that KDM5B binds further downstream into gene body regions compared with the distribution of H3K4me3 marks (Figure 1B). A more quantitative assessment of this phenomenon revealed an increased ratio of gene body to promoter methylation for KDM5B binding relative to H3K4me3 marks (Figure 1C), further demonstrating the occupancy of KDM5B in gene body regions. The profile of KDM5B binding is similar to enrichment of RNAPII and MLL4, which is a member of the mammalian H3K4 histone methyltransferase complexes, near TSSs (Figure 1D). Interestingly, KDM5B binds promoters of the core pluripotency regulators Oct4/*Pou5f1*, *Sox2*, and *Nanog* (Figure 1E), providing additional evidence that KDM5B positively supports ES cell self-renewal. A comparison of KDM5B binding sites with H3K4me3 islands revealed that >96% of KDM5B targets were enriched with H3K4me3 (Figure 1F, left Venn diagram). These results are in contrast to a previous study that showed KDM5B binds predominantly intragenic regions in ES cells [27], but are in alignment with a study that showed KDM5B binds active genes in human cells [33]. Because many developmental genes are marked by activating H3K4me3 and repressive H3K27me3 modifications in ES cells [34], we further compared KDM5B binding with H3K27me3-marked genes and bivalent genes marked by H3K4me3 and H3K27me3 [35]. Our results show that KDM5B co-localizes with 83% of H3K27me3 occupied promoters (Figure 1F, middle Venn diagram) and 93% of bivalent genes (Figure 1F, right Venn diagram). KDM5B co-localizes with H3K4me3 and H3K27me3 at promoters of bivalent developmental genes such as HoxA cluster genes (Figure 1G). Overall, these results demonstrate that KDM5B occupies active genes marked by H3K4me3, including core pluripotency-associated genes, and bivalent genes marked by H3K4me3 and H3K27me3 in ES cells.

A further comparison of KDM5B binding profiles with published ChIP-Seq datasets [3,35-39] revealed a strong correlation between KDM5B binding and ES cell-enriched TFs and histone modifications that associate with active genes in ES cells (Figure 1H). Although KDM5B binds 83% of H3K27me3 occupied promoters in ES cells (Figure 1F), because this analysis compares promoter occupancy of all genes in ES cells, KDM5B binding was not found to be correlated with polycomb complex proteins and H3K27me3. These results suggest that KDM5B binding is highly correlated with H3K4me3 marks at both active genes (for example, pluripotency regulators) and bivalent genes. These results also show that KDM5B binding is not correlated with H3K9me2 marks, which are associated with inactive genes in ES cells, suggesting that KDM5B targets mainly active genes in ES cells. We further evaluated co-binding of KDM5B and OCT4, SOX2, and NANOG and observed a significant number of genes were co-bound by OCT4, SOX2, and NANOG (Figure 1I).

### KDM5B prevents spreading of H3K4 methylation to gene bodies

**Figure 2:**
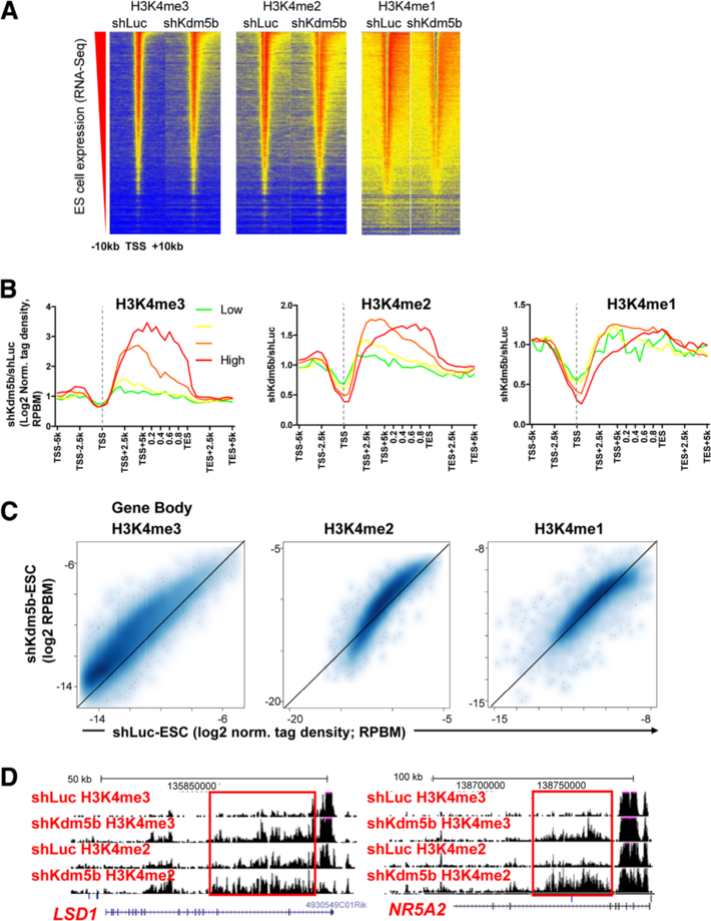
**KDM5B regulates H3K4 methylation at gene body regions.** Knockdown of *Kdm5b* transcripts results in altered H3K4 methylation profiles at promoters and enhancers and within gene body regions. **(A)** Heat map density of ChIP-Seq data at refseq genes sorted according to their absolute expression in ES cells. shKdm5b ES cells exhibit increased H3K4me3/2 methylation in gene body regions and decreased H3K4me3/2/1 methylation at promoter regions. **(B)** Correlation between changes in gene body histone methylation and expression level. Log2 fold change normalized tag density ratios (shKdm5b/shLuc) of H3K4me3, H3K4me2, and H3K4me1 at all refseq genes, which were sorted into four groups based on their absolute expression level in ES cells (red line, highest 25% expressed; green line, lowest 25% expressed). **(C)** Scatter plot of gene body densities of H3K4me3/2/1 in shLuc and shKdm5b ES cells. **(D)** UCSC genome browser examples of altered profiles of H3K4me3/2 within gene bodies of pluripotency-related genes such as *Lsd1* and *Nr5a2* in shKdm5b ES cells.

Because KDM5B is predominantly localized near promoters and gene body regions (Figure 1A-C), we evaluated the regulation of H3K4 methylation profiles at these sites by KDM5B. Interestingly, our results showed a marked increase in global gene body methylation (H3K4me3/2) in shKdm5b ES cells as shown in heat maps (Figure 2A) and average profiles (>10-fold for top 25% of H3K4me3, >3-fold for top 25% of H3K4me2) (Figure 2B), which is correlated with gene activity (red line indicates highest expressed genes; green line indicates lowest expressed genes). A comparison of H3K4 methylation densities of the top 13 k expressed genes in ES cells confirmed that *Kdm5b* knockdown leads to increased H3K4me3/2 levels in gene bodies of active genes (Figure 2C), such as LSD1/*Kdm1a* and *Nr5a2* (Figure 2D).

**Figure 3:**
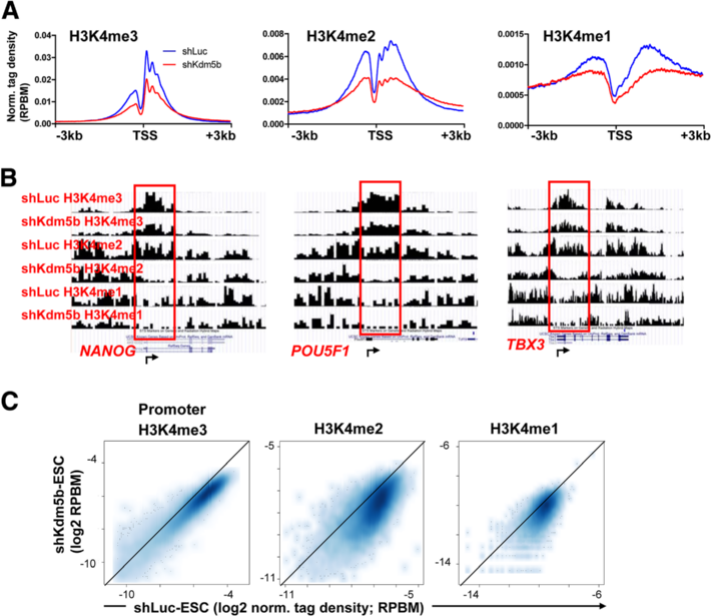
**KDM5B regulates H3K4 methylation at promoters. (A)** Log2 normalized tag density ratios (reads per base pair per million reads (RPBM)) of H3K4me3, H3K4me2, and H3K4me1 at TSSs of all refseq genes. **(B)** Browser view of altered profiles of H3K4me3/2/1 at promoters of genes such as *Nanog*, *Pou5f1*, and *Tbx3*. **(C)** Scatter plot of ChIP-Seq tag densities of H3K4me3/2/1 peaks in promoter regions in shLuc and shKdm5b ES cells.

We also observed decreased promoter methylation (H3K4me3/2/1) at active genes in shKdm5b ES cells (Figure 3A). Inspection of ChIP-Seq tracks revealed decreased promoter methylation at pluripotency regulators, including *Nanog*, Oct4/*Pou5f1*, and *Tbx3* (Figure 3B). Scatter plots also highlight decreased H3K4me3/2/1 densities of highly expressed genes (Figure 3C). Overall, these observations implicate a role for KDM5B in regulating the localization of H3K4 methylation marks at active genes in ES cells.

### Spreading of H3K4me3 to gene bodies leads to defects in gene expression in ES cells

**Figure 4:**
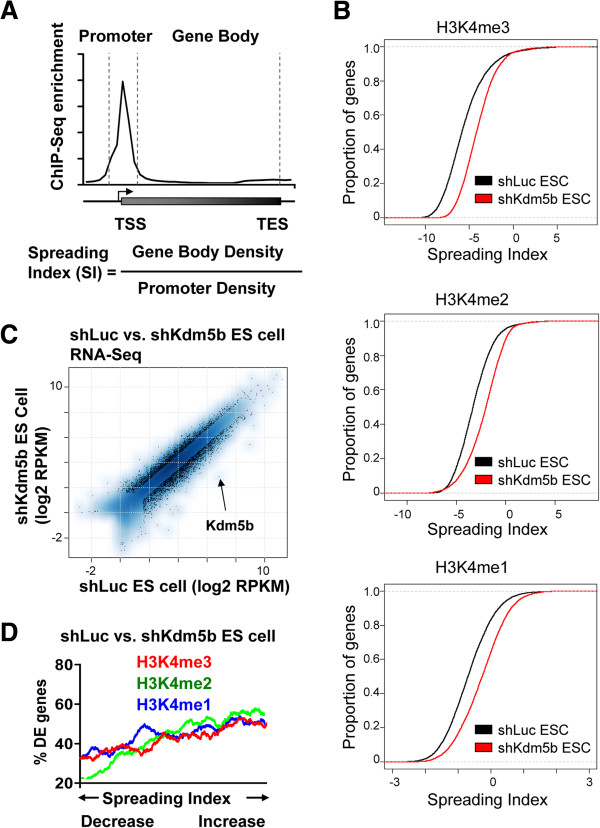
**Spreading of H3K4me3 to gene bodies leads to defects in gene expression in ES cells. (A)** Schematic representation describing the calculation used to determine the SI of genes marked by H3K4 methylation. The promoter bin is defined as a 1 kb, 1.5 kb, or 3 kb window around the TSS of genes marked by H3K4me3, H3K4me2, and H3K4me1, respectively, while the transcribed region (gene body) is defined as the region extending to the TES. The SI is calculated from the ratio of the density of H3K4 methylation in the gene body bin to the density of H3K4 methylation in the promoter bin. **(B)** Empirical cumulative distribution for the SI of H3K4me3 (top panel), H3K4me2 (middle panel), and H3K4me1 (bottom panel) across all genes for shLuc (black) and shKdm5b (red) ES cells. Y-axis shows the percentage of genes that exhibit a SI less than the value specified by the x-axis. A line shifted to the right means a systematic increase in the spreading index. *P*-value for all <1E-5 (Kolmogorov-Smirnov test). Note the increased SI for genes marked by H3K4me3, H3K4me2, and H3K4me1 in shKdm5b ES cells. **(C)** Scatter plot of gene expression measured by RPKM (reads per kilobases of exon model per million reads). Log2-adjusted differentially expressed (DE) genes (>1.5-fold; false discovery rate <0.001; RPKM >11) are shown in black. **(D)** Spreading of H3K4 methylation into gene bodies is associated with defects in gene expression in *Kdm5b* knockdown ES cells. Genes are ordered on the x-axis from left to right based on the fold change in their SI (shKdm5b/shLuc; ES cells) from low to high. A sliding window of 1,000 genes was used to calculate the percentage of DE genes (y-axis). The analysis was performed independently using SIs calculated for H3K4me3 (red), H3K4me2 (green), and H3K4me1 (blue) marks.

Previously, we showed that KDM5B is important for ES cell differentiation, where depletion of KDM5B results in an extension of self-renewal under differentiation conditions, persistent expression of self-renewal genes, and delayed expression of bivalent developmental genes [32]. To investigate whether the redistribution of H3K4 methylation is linked to defects in gene expression in KDM5B-depleted ES cells, we quantified the distribution of H3K4 methylation by calculating the relative ratio of H3K4 methylation in gene bodies to that in promoter regions, which we have termed the ‘spreading index’ (SI) (Figure 4A). Using this calculation, we observed an increase in the SI for genes marked by H3K4me3, H3K4me2, or H3K4me1 in KDM5B-depleted ES cells (red) relative to control ES cells (black) (Figure 4B). An investigation of the relationship between spreading of H3K4 methylation and gene expression in shLuc versus shKdm5b ES cells (Figure 4C) revealed that genes with more spreading of H3K4 methylation into gene bodies are more sensitive to transcriptional dysregulation in *Kdm5b* knockdown ES cells (Figure 4D), suggesting that a redistribution of H3K4 methylation leads to dysregulation of gene expression in KDM5B-depleted ES cells.

### KDM5B prevents spreading of H3K4 methylation to enhancer shores

**Figure 5:**
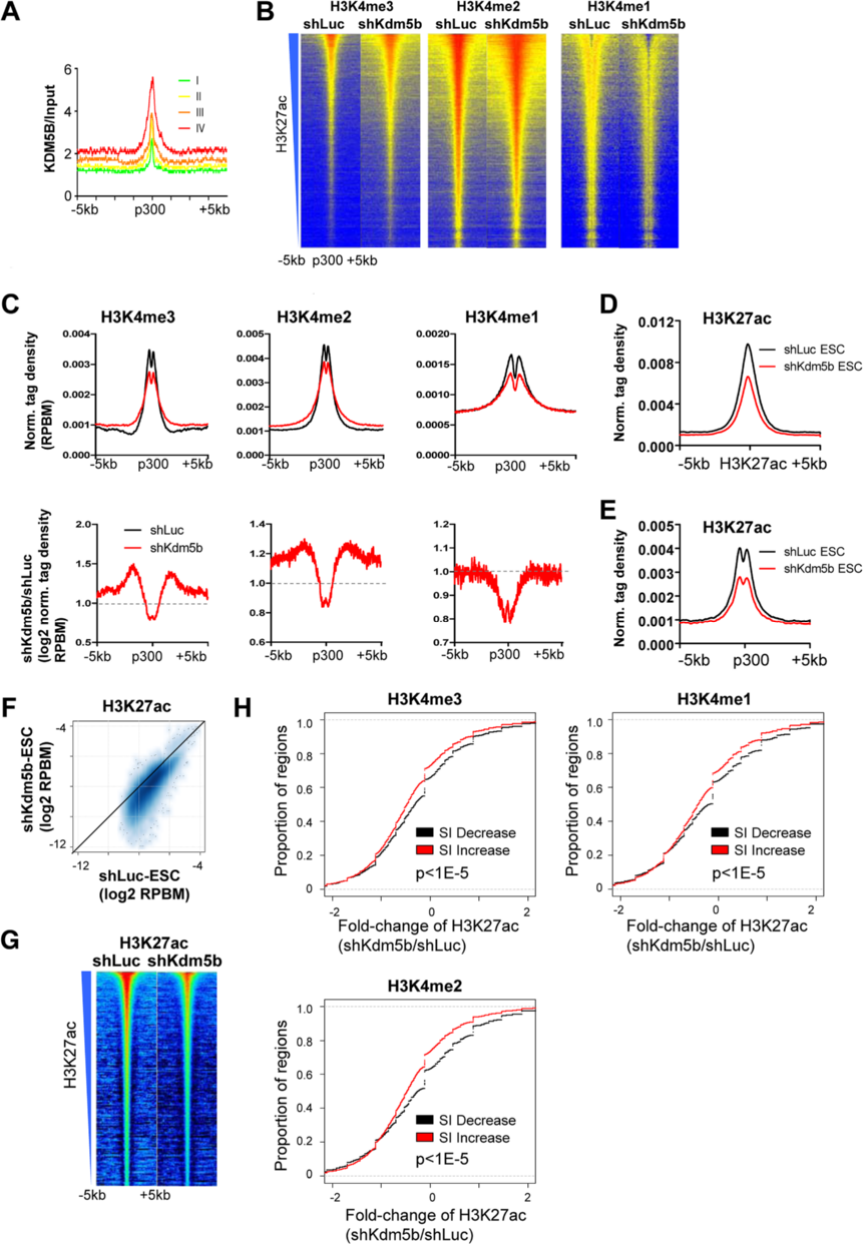
**KDM5B controls enhancer activity by regulating H3K4 methylation. (A)** KDM5B binding at p300 enhancer sites sorted by enrichment level of acetylated H3K27 (H3K27ac). **(B)** Heat map density profiles of H3K4me3/2/1 around intergenic p300 binding sites, sorted by H3K27ac levels. **(C)** Top: average profile of H3K4me3/2/1 density at intergenic p300 sites in shLuc and shKdm5b ES cells. Bottom: log2 fold change normalized tag density ratios (shKdm5b/shLuc) of H3K4me3, H3K4me2, and H3K4me1 at p300 enhancers. RPBM, reads per base pair per million reads. **(D)** Average profiles of H3K27ac tag densities around H3K27ac peaks in shLuc and shKdm5b ES cells. **(E)** Average profiles of H3K27ac tag densities of H3K27ac around p300 binding sites (proxies of enhancers) in shLuc and shKdm5b ES cells. **(F)** Scatter plot of H3K27ac tag densities in shLuc and shKdm5b ES cells. **(G)** Heat map density profiles of H3K27ac peaks, sorted by H3K27ac levels. **(H)** Spreading of H3K4 methylation into enhancer shores in *Kdm5b* knockdown ES cells is associated with a decrease in H3K27ac levels. Shown are empirical cumulative distributions for the change in H3K27ac (shKdm5b/shLuc) across two groups of enhancers sorted by changes in spreading indices, calculated independently for H3K4me3 (left panel), H3K4me2 (middle panel) or H3K4me1 (right panel). Y-axis shows the percentage of enhancers that exhibit a change in H3K27ac density less than the value specified by the x-axis. A line shifted to the left means a systematic decrease in H3K27ac levels. *P*-value for all <1E-5 (Kolmogorov-Smirnov test).

Next, we investigated whether KDM5B also occupies enhancer regions by examining KDM5B signals at p300 binding sites, reported by others as proxies for enhancers [41], that were sorted according to their levels of H3K27ac, which marks active enhancers [42,43]. These results show that KDM5B occupies active enhancers (Figure 5A; red line, top 25% H3K27ac; green line, bottom 25%). Concomitant with changes observed for promoter and gene body H3K4 methylation levels following knockdown of *Kdm5b* in ES cells, regions immediately adjacent to p300-bound regions, or p300 enhancer shores, also contained modestly increased H3K4me3/2 and decreased H3K4me1 levels, as evident in heat maps (Figure 5B) and average profiles (Figure 5C), further suggesting that KDM5B regulates H3K4 methylation at enhancers. These results implicate a role for KDM5B in localizing H3K4 methylation at enhancers, thereby preventing its spreading to enhancer shores.

To determine whether spreading of H3K4 methylation to enhancer shores leads to altered enhancer activity, we surveyed H3K27ac levels, a hallmark of enhancer activity [42-44], at p300 binding sites in shLuc and shKdm5b ES cells. We observed a decrease in acetylated lysine 27 of histone 3 (H3K27ac) levels at H3K27ac peaks (Figure 5D) and at intergenic p300 peaks (Figure 5E) in KDM5B-depleted ES cells, as evident in average profiles (Figure 5D,E). A scatter plot (Figure 5F) and heat maps (Figure 5G) also reveal decreased H3K27ac densities in KDM5B-depleted ES cells. Moreover, an investigation of the relationship between spreading of H3K4 methylation and enhancer activity revealed that enhancers with spreading of H3K4 methylation into shores in KDM5B-depleted ES cells exhibited greater decreases in H3K27ac levels (Figure 5H). Overall, these findings demonstrate that spreading of H3K4 methylation to enhancer shores leads to decreased enhancer activity, thus implicating a role for KDM5B in regulating enhancer activity in ES cells.

### KDM5B regulates H3K4 methylation during ES cell differentiation

**Figure 6:**
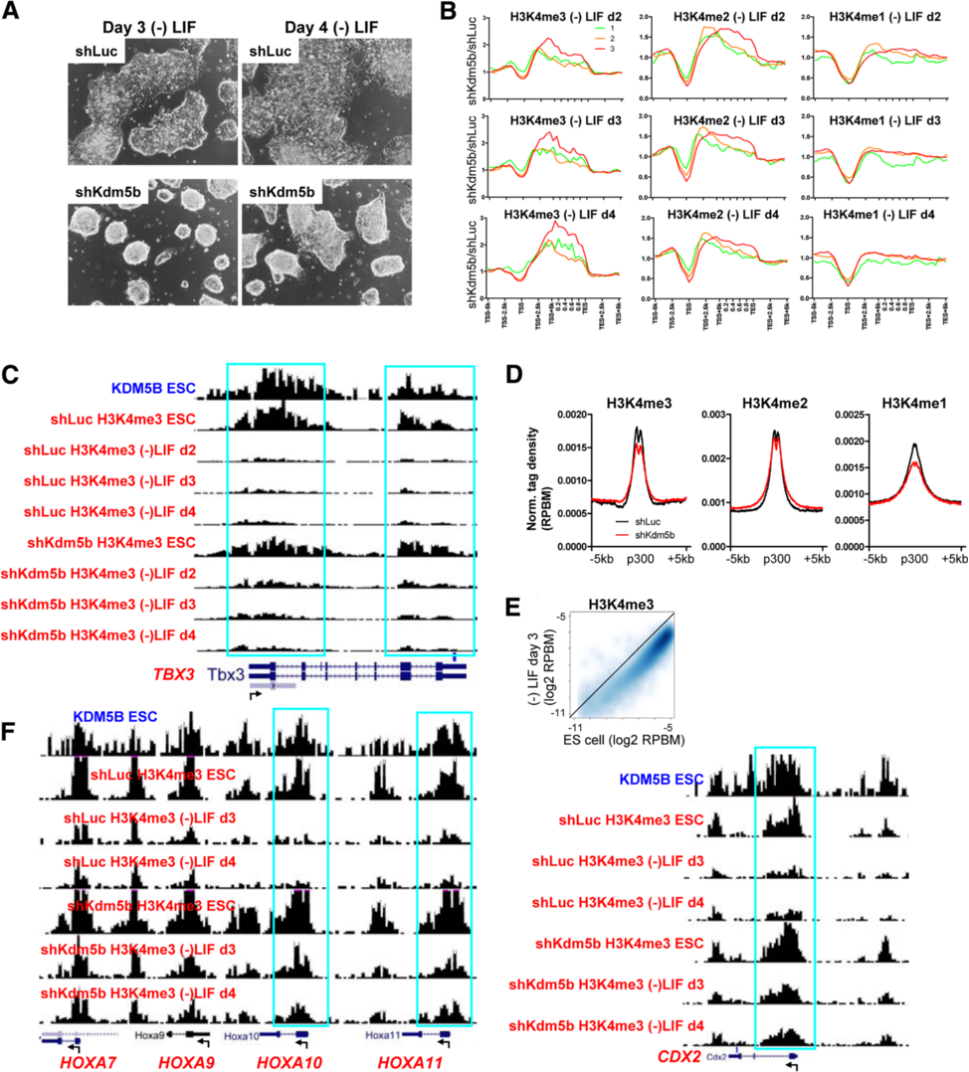
**KDM5B regulates H3K4 methylation during differentiation. (A)** ES cells cultured in the absence of LIF for 3 to 4 days to induce differentiation. Note the loss of normal three-dimensional colony morphology of shLuc ES cells at day 3 of differentiation, while shKdm5b ES cells maintained their normal three-dimensional colony morphology in the absence of LIF. **(B)** Correlation between changes in histone methylation and expression level. Fold change normalized tag density ratios (shKdm5b/shLuc) of H3K4me3, H3K4me2, and H3K4me1 at all refseq genes, which were sorted into three groups based on their expression level (red line, highest expressed; green line, lowest expressed). **(C)** UCSC genome browser view of H3K4me3/2/1 marks at the self-renewal gene *Tbx3* during differentiation. shKdm5b ES cells have altered H3K4me3/2/1 levels relative to shLuc ES cells. **(D)** Average profile of H3K4me3/2/1 densities at p300 enhancer regions following 4 days of shLuc and shKdm5b ES cell differentiation. RPBM, reads per base pair per million reads. **(E)** Promoter density of H3K4me3 in ES cells and day 3 differentiated ES cells. **(F)** UCSC browser view of H3K4me3 density at HoxA cluster genes during differentiation. Note the delayed decrease of H3K4me3 levels during shKdm5b ES cell differentiation relative to shLuc ES cells.

Previous studies have shown that KDM5B is important for differentiation [25,26,30]. It is possible that the differentiation defect of KDM5B-depleted cells is due to misregulation of histone methylation during early differentiation. To test this possibility, we differentiated shKdm5b ES cells by culturing in the absence of LIF over a time-course of four days. While shLuc ES cell colonies became flattened following removal of LIF, indicative of differentiation, shKdm5b ES cells remained in a tight three-dimensional colony formation, suggesting a resistance to changes in cellular fate, or maintenance of self-renewal under differentiation-inducing conditions (Figure 6A). We then used ChIP-Seq to interrogate global H3K4 methylation profiles during ES cell differentiation. These results demonstrate that shKdm5b ES cells exhibit decreased promoter H3K4 methylation and increased gene body H3K4 methylation at highly expressed ES cell genes during differentiation (Figure 6B). This pattern is consistent with the effects of *Kdm5b* knockdown on H3K4 methylation profiles in ES cells under self-renewing conditions. While H3K4 methylation was downregulated at promoters of most self-renewal genes (for example, *Pou5f1*, *Nanog*) during shKdm5b ES cell differentiation, H3K4 methylation was not equally downregulated at several self-renewal genes, such as *Tbx3* (Figure 6C), relative to shLuc ES cells, which may in part explain the delayed differentiation of shKdm5b ES cells. KDM5B binding in ES cells overlaps with H3K4 methylation changes observed during differentiation (Figure 6C, top track). However, because shKdm5b ES cells exhibit depleted H3K4 methylation at promoter regions under self-renewing conditions, and continue to self-renew, albeit at a reduced pace, changes in global promoter H3K4 methylation may not alone explain the delayed differentiation ability of shKdm5b ES cells.

To further understand epigenetic changes resulting from *Kdm5b* knockdown, we evaluated H3K4 methylation at p300 enhancers during ES cell differentiation. Interestingly, p300-bound enhancers showed similar patterns of H3K4 methylation during differentiation (day 4) relative to ES cells grown under self-renewing conditions (Figure 6D), which is also consistent with the trend observed at promoter regions. Because ES cell differentiation is a dynamic process involving epigenetic alterations to chromatin and transcriptional changes, to further understand the role of KDM5B in differentiation we surveyed changes in H3K4 methylation at key developmental genes during shKdm5b ES cell differentiation. Interestingly, promoter H3K4me3 levels decreased on a global level following three days of ES cell differentiation (Figure 6E), which has also been observed in other recent ES cell time-course differentiation reports [45,46]. However, knockdown of *Kdm5b* led to persistence of H3K4me3 signals at a subset of genes bound by KDM5B in ES cells (Figure 6F, top tracks), including HoxA cluster genes and other developmental genes such as *Cdx2* (Figure 6F) during three to four days of ES cell differentiation.

**Figure 7:**
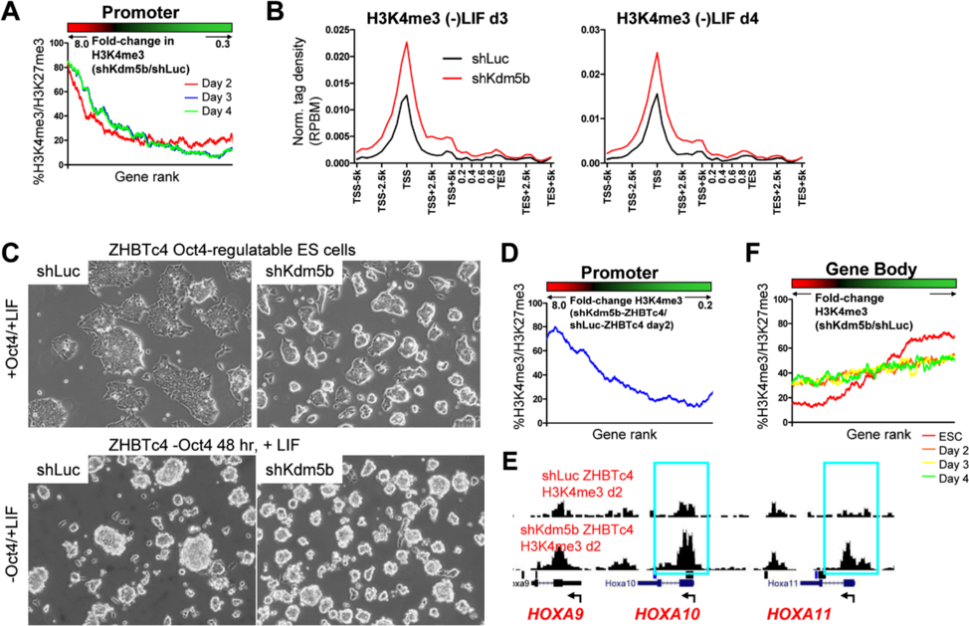
**KDM5B regulates H3K4 methylation at bivalent genes during differentiation without LIF and Oct4. (A)** Relationship between changes in promoter H3K4me3 density during differentiation and the percentage of genes with bivalent marks (H3K4me3/H3K27me3), using a sliding window of 500 genes (red, increased H3K4me3; black, no-change in H3K4me3; green, decreased H3K4me3). **(B)** Average profile of H3K4me3 density at genes with increased H3K4me3 during shKdm5b differentiation. RPBM, reads per base pair per million reads. **(C)**  Oct4-regulatable ES cells (ZHBTc4) infected with shLuc or shKdm5b lentiviral particles were **(D)** cultured in the presence of doxycycline to downregulate OCT4 levels and induce differentiation. Relationship between changes in promoter H3K4me3 density during differentiation in the absence of OCT4 and KDM5B and the percentage of genes with bivalent marks (red, increased H3K4me3; black, no-change in H3K4me3; green, decreased H3K4me3). **(E)** Browser view of H3K4me3 density following differentiation of shKdm5b ZHBTc4 ES cells. **(F)** Relationship between changes in gene body H3K4me3 density during differentiation and the percentage of genes with bivalent marks.

To investigate whether KDM5B particularly impacts H3K4me3 at bivalent genes (marked by H3K4me3/H3K27me3) in differentiated shKdm5b ES cell, we ranked genes according to their change in H3K4me3 level within promoter regions following knockdown of *Kdm5b* and evaluated the percentage of bivalent genes using a sliding window of 500 genes (Figure 7A). The analysis revealed that a greater percentage of bivalent genes exhibited increased H3K4me3 following shKdm5b ES cell differentiation relative to genes with decreased H3K4me3 levels (Figure 7A), suggesting that KDM5B plays a critical role in erasing H3K4me3 at bivalent genes during ES cell differentiation. The TSS-TES profiles of genes with increased H3K4me3 are shown in Figure 7B. To confirm these results in another differentiation context, we forced the differentiation of ES cells by depleting OCT4 levels using the Oct4-regulatable ES cell line ZHBTc4 [47] in combination with RNA interference-mediated reduction of *Kdm5b* transcripts as described above, and subsequently performed ChIP-Seq to evaluate global H3K4me3 levels. In the presence of OCT4 and LIF, the phenotype of shKdm5b ZHBTc4 ES cells relative to shLuc ZHBTc4 ES cells is similar to the phenotype of KDM5B-depleted ES cells as described above, where shKdm5b ZHBTc4 ES cells have a slightly reduced colony size (Figure 7C, top). Following two days of OCT4 depletion in the presence of doxycycline (Figure 7C, bottom), we observed global decreases in H3K4me3 levels, which was similar to our previous results following differentiation in the absence of LIF. Moreover, we also observed a greater percentage of bivalent genes, including Hox genes, with elevated H3K4me3 levels following differentiation of shKdm5b ZHBTc4 ES cells (Figure 7D,E). To understand if this pattern is unique to promoter regions, we also investigated changes in H3K4me3 levels within gene body regions of bivalent genes in shKdm5b ES cells. Interestingly, while we observed a greater percentage of bivalent genes with decreased H3K4me3 in gene bodies prior to differentiation, this differential methylation decreased somewhat during differentiation (Figure 7F), suggesting that gene body methylation is not as resistant to changes in H3K4me3 levels at bivalent genes relative to promoter regions. Because expression of self-renewal genes is normally discontinued during differentiation, demethylation of gene body regions is not likely to play a predominant role in turning over expression programs relative to promoter deactivation. Overall, these results suggest that KDM5B demethylates bivalent genes during differentiation, where ES cells lacking KDM5B are unable to efficiently reset the self-renewal epigenetic landscape and adequately establish a new expression paradigm to facilitate differentiation.

### Spreading of H3K4me3 to gene bodies leads to defects in gene expression during ES cell differentiation

Our results demonstrate that KDM5B occupies bivalent genes in ES cells, and depletion of KDM5B leads to underexpression of bivalent developmental genes during ES cell differentiation [32], spreading of H3K4 methylation to gene bodies during differentiation, and persistent H3K4me3 marks at bivalent genes during differentiation.

**Figure 8:**
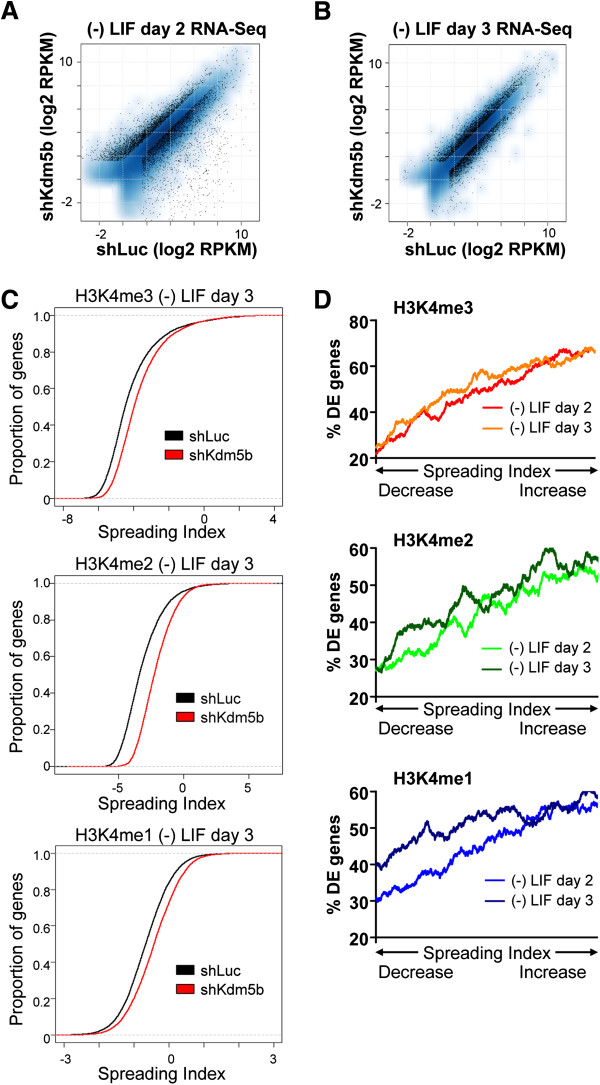
**Spreading of H3K4me3 to gene bodies leads to defects in gene expression during ES cell differentiation.** Scatter plot of gene expression measured by RPKM for shLuc and shKdm5b ES cells differentiated in the absence of LIF for **(A)** 48 h (day 2) and **(B)** 72 h (day 3). Log2-adjusted differentially expressed genes (>1.5-fold; FDR <0.001; RPKM >1) are shown in black. **(C)** Empirical cumulative distribution for the spreading index of H3K4me3 (top panel), H3K4me2 (middle panel), and H3K4me1 (bottom panel) across all genes for shKdm5b knockdown cells (red line) and shLuc control cells (black line) following differentiation for three days after LIF withdrawal. Y-axis shows the percentage of genes that exhibit a spreading index less than the value specified by the x-axis. A line shifted to the right means a systematic increase in the spreading index. *P*-value for all <1E-5 (Kolmogorov-Smirnov test). **(D)** Spreading of H3K4me into gene body regions is associated with defects in gene expression during differentiation of KDM5B-depleted ES cells. Genes were ordered on the x-axis from left to right based on the fold change in their SI (shKdm5b/shLuc) from low to high. A sliding window of 1,000 genes was used to calculate the percentage of differentially expressed (DE) genes (y-axis) in KDM5B-depleted cells. The analyses were performed independently using SIs calculated from H3K4me3 (top panel), H3K4me2 (middle panel), and H3K4me1 (bottom panel) marks following two and three days of ES cell differentiation after LIF withdrawal.

To investigate a correlation between changes in H3K4 methylation levels at gene body and promoter regions, and gene expression changes during differentiation of KDM5B-depleted ES cells, we first used RNA-Seq data to identify differentially expressed genes (fold change >1.5; false discovery rate (FDR) <0.001; RPKM (reads per kilobases of exon model per million reads) >1) during differentiation in the absence of KDM5B for two days (48 h; Figure 8A) and three days (72 h; Figure 8B). We then characterized H3K4 methylation in gene body and promoter regions using the SI (Figure 4A). Using this metric, we observed an increase in the SI for genes marked by H3K4me3, H3K4me2, or H3K4me1 following three days of differentiation of KDM5B-depleted ES cells (red) relative to control ES cells (black) (Figure 8C). An investigation of the relationship between spreading of H3K4 methylation and gene expression revealed that genes with greater spreading of H3K4 methylation into gene bodies are more sensitive to transcriptional dysregulation during differentiation of *Kdm5b* knockdown ES cells (Figure 8D). These findings suggest that a redistribution of H3K4 methylation leads to dysregulation of gene expression during differentiation of KDM5B depleted ES cells.

### KDM5B and LSD1 co-regulate H3K4 methylation in ES cells

**Figure 9:**
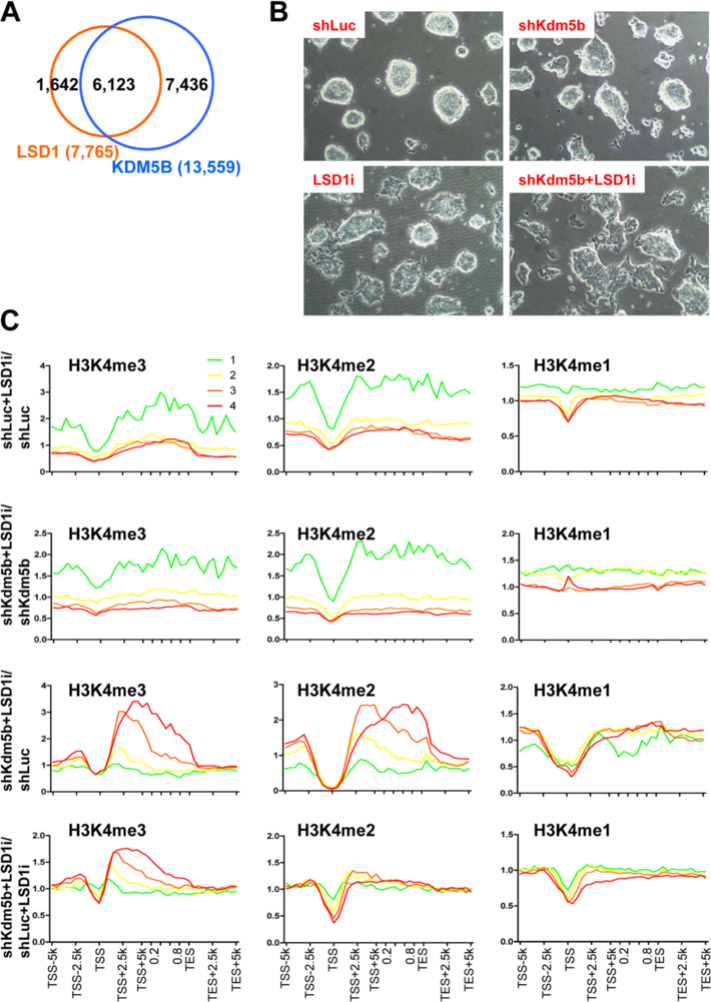
**KDM5B and LSD1 co-regulate H3K4 methylation in ES cells. (A)** Venn diagram showing overlap of KDM5B- and LSD1-bound genes in ES cells. **(B)** Bright field microscopy of shLuc and shKdm5b ES cells cultured in the presence of 2 μM of the LSD1 inhibitor tranylcypromine/parnate (LSD1i) for 4 days. **(C)** Correlation between changes in histone methylation and expression level. Fold change normalized tag density ratios of H3K4me3, H3K4me2, and H3K4me1 at refseq genes were sorted into four groups based on their absolute expression level. Note that the lowest expressed genes have increased H3K4me3/2/1 levels in LSD1i-treated ES cells (red line, highest 25% expressed; green line, lowest 25% expressed).

**Figure 10:**
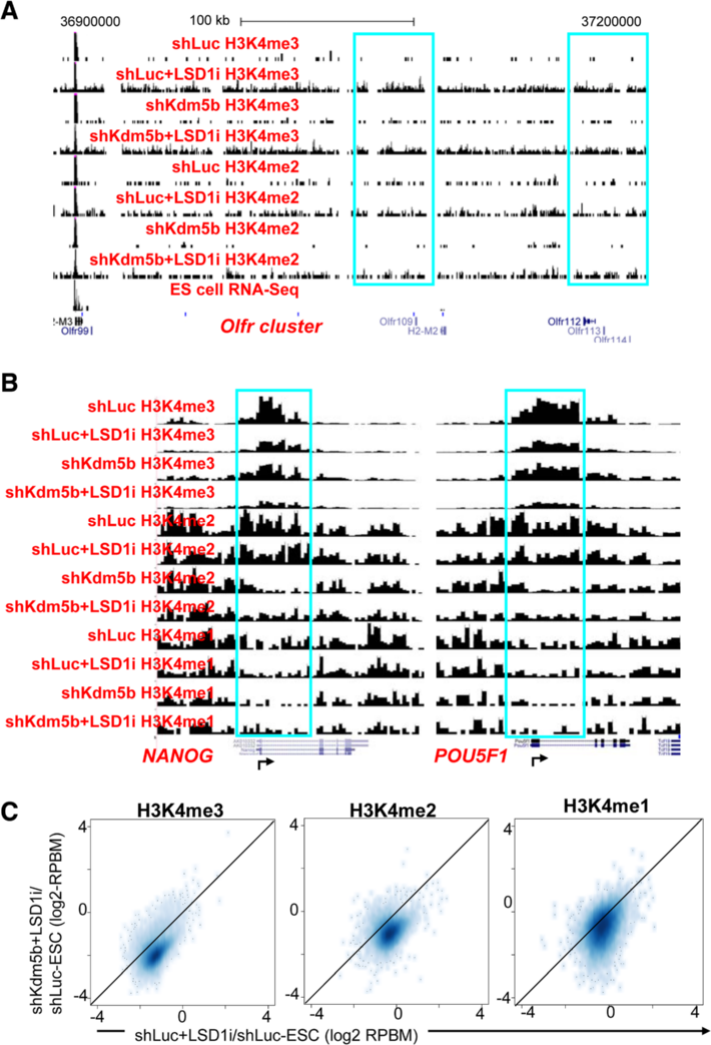
**H3K4 methylation profiles in KDM5B-depleted and LSD1-inhibited ES cells.** Browser views of H3K4me3/2/1 densities at **(A)** olfactory receptor and **(B)** pluripotency genes (*Nanog*, *Pou5f1*). **(C)** Fold change densities of H3K4me3/2/1 peaks in shLuc?+?LSD1i and shKdm5b?+?LSD1i ES cells relative to shLuc ES cells. RPBM, reads per base pair per million reads.

In addition to KDM5B, LSD1 (KDM1A) also regulates H3K4 methylation [21] and critically contributes to embryo development and ES cell pluripotency [48-51]. To understand the respective function of KDM5B and LSD1 in regulating ES cell chromatin, we compared the genome-wide binding of KDM5B and LSD1 [39] and found that they co-occupy 6,123 genes in ES cells (Figure 9A), which comprises 79% of LSD1 targets and 45% of KDM5B targets. To investigate the function of LSD1 in regulating H3K4 methylation, we treated ES cells with the LSD1 inhibitor tranylcypromine (parnate/TCP (LSD1i)) (Figure 9B), and evaluated the global distribution of H3K4 methylation using ChIP-Seq (Figure 9C). Although treatment with LSD1i resulted in modest changes in cellular morphology (Figure 9B), inhibition of LSD1 resulted in decreased H3K4 promoter methylation of highly expressed genes and increased H3K4 methylation in gene body regions of lowly expressed genes as shown in average profiles (>5-fold for bottom 25% of H3K4me3, >3-fold for bottom 25% of H3K4me2) (Figure 9C). Increased gene body methylation is also visible in genome tracks of H3K4 methylation at lowly expressed olfactory receptor genes (Figure 10A). Moreover, a combined inhibition of LSD1 and *Kdm5b* knockdown resulted in greater decreases in H3K4me3/2/1 levels at active promoters (for example, *Nanog*, *Pou5f1*) relative to treatment with LSD1i or *Kdm5b* knockdown alone (Figure 10B,C). These differences are evident in average profile graphs (Figure 9C) and browser views (Figure 10B), where H3K4me2 has been reduced to near baseline upon LSD1 inhibition and *Kdm5b* knockdown, and density scatter plots (Figure 10C). However, simultaneous inhibition of LSD1 and *Kdm5b* knockdown did not augment H3K4 methylation levels in gene body regions, which we observed in shKdm5b ES cells, suggesting that LSD1 and KDM5B serve unique functions in ES cells. While both KDM5B and LSD1 regulate H3K4 methylation levels at promoters, our results demonstrate that KDM5B demethylates gene body regions of active genes while LSD1 demethylates gene body regions of inactive genes in ES cells.

**Figure 11:**
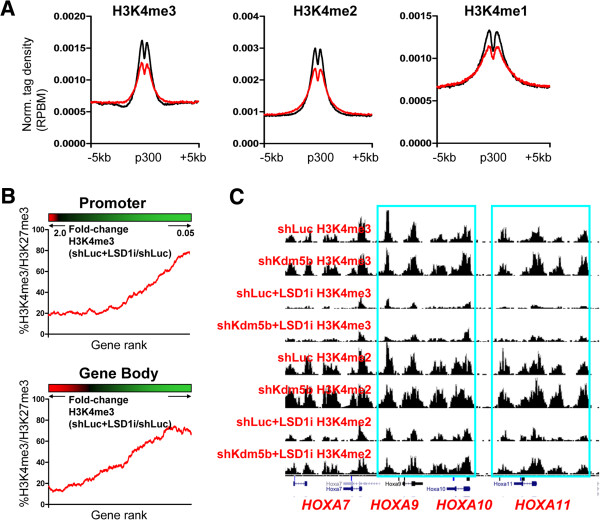
**KDM5B and LSD1 regulate H3K4 methylation at promoters and enhancers in ES cells. (A)** Average profile of H3K4me3/2/1 density at p300 enhancer regions in LSD1i-treated ES cells (red line) relative to shLuc ES cells (black line). RPBM, reads per base pair per million reads. **(B)** Relationship between changes in promoter and gene body H3K4me3 density and the percentage of genes with bivalent marks (red, increased H3K4me3; black, no-change in H3K4me3; green, decreased H3K4me3). **(C)** Genome browser view of H3K4me3 levels at HoxA cluster bivalent genes upon inhibition of LSD1 in ES cells.

We also observed altered H3K4 methylation levels at enhancer regions marked by p300 binding upon inhibition of LSD1 (Figure 11A). Inhibition of LSD1 resulted in decreased H3K4me3/2/1 levels at p300 enhancers, while enhancer shores showed modest increases in H3K4me3/2 levels, suggesting that KDM5B and LSD1 both regulate H3K4 methylation at enhancer regions. Intriguingly, we found decreased H3K4 methylation levels at bivalent genes in LSD1i ES cells, including HoxA cluster genes, which is in contrast to the elevated H3K4 methylation in the *Kdm5b* knockdown cells. By ranking genes according to their change in H3K4me3 level (shLuc LSD1i versus shLuc) within promoter regions and evaluating the percentage of bivalent genes using a sliding window of 500 genes, we observed a greater percentage of bivalent genes with decreased H3K4me3 following LSD1 inhibition relative to genes with increased H3K4me3 levels (Figure 11B), suggesting that bivalent genes are unable to maintain H3K4 methylation levels following inhibition of LSD1. Specifically, we observed decreased H3K4me3 levels at Hox genes (Figure 11C). In summary, these results suggest distinct functional roles for KDM5B and LSD1 at bivalent genes, where KDM5B is dispensable for maintaining H3K4 methylation at bivalent genes while LSD1 is involved in maintaining H3K4 methylation in ES cells.

## Discussion

### KDM5B regulates H3K4 methylation in gene bodies and enhancer shores

Epigenomic analysis of KDM5B binding and H3K4 methylation upon depletion of KDM5B revealed its global targets and core function. We found that KDM5B binds to promoters and gene bodies of active genes, enhancers, and co-localizes with H3K4me3 marks and core pluripotency TFs (Figure 1H). As expected, *Kdm5b* knockdown led to a net gain of H3K4me3/2 as assessed by western blotting [32], although we did not observe significant changes in the global levels of H3K4me1 in *Kdm5b* knockdown ES cells (Additional file 1). However, *Kdm5b* knockdown led to a striking redistribution of H3K4 methylation, including global increases within gene body regions (Figure 2) and decreases in promoters of highly active genes (Figure 3), which were correlated with changes in gene expression in ES cells (Figure 4D). There are several possible explanations for this phenomenon. First, our data show that KDM5B binding is highly enriched at TSS regions (Figure 1A), but KDM5B binds further downstream into gene body regions compared with the distribution of H3K4me3 marks (Figure 1B). KDM5B may prevent spreading of H3K4 methylation to gene body regions by resetting H3K4 methylation after each transcriptional cycle, or upon gene activation, which has been suggested previously in a different context for histone deacetylase activity [52,53]. An inability to properly reset H3K4 methylation levels may lead to skewed H3K4 methylation profiles, including decreased levels at promoters. To facilitate gene body demethylation, KDM5B may associate with unphosphorylated RNAPII, initiated RNAPII (Ser5P), or elongating RNAPII (Ser2P) in a manner analogous to the association of components of the MLL methyltransferase complex, WDR5 and ASH2L, with RNAPII Ser2P or Ser5P, respectively [27]. Second, activity of other H3K4 demethylases with partially overlapping or redundant functions may compensate for the depletion of KDM5B, leading to decreased H3K4 demethylation at promoters. While we did not observe differential expression of other demethylases in the absence of KDM5B, our data show that a combined depletion of KDM5B and inhibition of LSD1 in ES cells leads to greater decreases in H3K4me3/2 levels at promoters compared with individual perturbation of *Kdm5b* or LSD1 (Figure 10B,C). Third, KDM5B may associate with H3K4 methyltransferases to reinforce their activity or promote their recruitment to promoter regions. In this case, depletion of KDM5B may lead to decreased promoter H3K4 methylation and aberrant H3K4 methlyation in gene bodies due to deficiencies in recruitment or activity. Fourth, KDM5B-mediated demethylation of promoter-adjacent regions may allow for focused, or sharp, H3K4 methylation peaks near TSS regions, thus serving as a platform for RNAPII binding [9,54] and recognition by H3K4 methyltransferases (for example, MLL/trxG proteins). In the absence of KDM5B, H3K4 methylation spreading to gene bodies may lead to aberrant H3K4 methylation by methyltransferases as a result of a diffuse H3K4 methylation platform to target.

Our finding that KDM5B demethylates gene body regions is partially in alignment with a previous study suggesting that KDM5B removes local domains of intragenic H3K4me3 [27]. However, while we observed global increases in H3K4 methylation (H3K4me3, H3K4me2) in gene body regions of active genes, including pluripotency regulators, in KDM5B-depleted ES cells, it is unclear from the Xie *et al*. study [27] whether depletion of KDM5B leads to global changes in H3K4 methylation levels. Also, Xie *et al*. suggest that KDM5B binds predominantly intragenic regions, which is in contrast to our findings and results presented in another previous study examining KDM5B binding in human cells [33], both of which demonstrate that KDM5B binding is highly correlated with H3K4me3 marks at active promoters.

We also observed similar changes in H3K4 methylation at enhancer sites compared with promoters following knockdown of *Kdm5b*, including decreased levels at p300 enhancer peaks and increased levels at neighboring regions, providing further evidence that KDM5B functions to focus H4K4 methylation. Our results also reveal that KDM5B-depleted ES cells have decreased enhancer activity, as measured by H3K27ac levels (Figure 5D-G), demonstrating that KDM5B is important in regulating enhancer function in ES cells. Increased H3K4 methylation in gene bodies and enhancer shores may support delayed differentiation by providing a broader platform for recognition by transcriptional machinery, thus reinforcing expression of self-renewal genes. It is likely that shKdm5b ES cells fail to properly differentiate due to a combination of inefficient deactivation of self-renewal genes and activation of differentiation genes.

### KDM5B regulates H3K4 methylation during differentiation

Our results also support a role for KDM5B in regulating H3K4 methylation at developmental genes during differentiation. Previous studies have shown that lineage-specific genes are underexpressed following EB differentiation of KDM5B-depleted ES cells [25-27]. These studies underscore the importance of KDM5B for differentiation. We found that shKdm5b ES cells retain higher levels of H3K4me3 at bivalent genes during differentiation compared to shLuc ES cells. The delayed differentiation of KDM5B-depleted ES cells may be due to the inability to efficiently remove H3K4 methylation marks at bivalent genes during differentiation, which may be required to reset the underlying epigenetic profile. While bivalent genes have been reported to maintain H3K4me3 marks at similar levels between ES cells and differentiated cells at key developmental genes [34,35,38], the levels of these marks during differentiation have not been fully addressed. We show that H3K4me3 levels initially decrease at self-renewal and bivalent genes during early differentiation. H3K4 methylation levels are then re-established upon lineage commitment. Our results describing global decreases in H3K4me3 during differentiation are supported by two recent studies [45,46]. The initial depletion of H3K4me3 at developmental genes during differentiation underscores an intriguing and underexplored regulatory aspect of this mark during lineage commitment. We also demonstrate that spreading of H3K4 methylation to gene bodies leads to differential gene expression during differentiation (Figure 8D). Altogether, these findings provide novel insights into the role of H3K4me3 marks at bivalent developmental genes during differentiation.

### Similarities and differences between KDM5B and LSD1

Our results demonstrate that KDM5B and LSD1 co-regulate H3K4 methylation at promoters of highly active genes, where knockdown of *Kdm5b* or inhibition of LSD1 resulted in decreased H3K4 methylation (Figure 3A-C, Figure 10B-C). Moreover, combined knockdown of *Kdm5b* and inhibition of LSD1 led to a near complete loss of H3K4me2 at promoters (Figure 9C, middle panel). However, H3K4me3/1 levels were not reduced to the same degree at these sites, suggesting that KDM5B and LSD1 serve distinct roles in regulating tri-, di-, and mono-methylation states at promoters. This is consistent with the respective substrate specificities of these enzymes, where KDM5B demethylates H3K4me3/2/1 while LSD1 demethylates H3K4me2/1.

KDM5B and LSD1 also differentially regulate H3K4 methylation at bivalent genes. While *Kdm5b* knockdown led to increased or unaltered H3K4me3 levels at bivalent genes in ES cells, inhibition of LSD1 resulted in decreased H3K4 methylation levels. KDM5B and LSD1 also differ in their regulation of H3K4 methylation within gene body regions, where KDM5B demethylates gene bodies of active genes (Figure 2) while LSD1 demethylates gene bodies of inactive genes (Figure 9C). Demethylation of gene bodies may serve to focus H3K4 methylation near promoters and enhancers, thus preventing the spread of methylation into surrounding nucleosomes. Alternatively, demethylation of gene body regions may also serve to reset nucleosome methylation after RNAPII mediated transcription.

## Conclusions

Data presented here provide novel insight into epigenomic regulation of H3K4 methylation in ES cells, where KDM5B functions to focus H3K4 methylation near promoters and enhancers to prevent the spread of these marks to surrounding regions.

## Materials and methods

### Embryonic stem cell culture

R1 ES cells, obtained from ATCC (Manassas, VA, USA), were cultured as previously described with minor modifications [55]. Briefly, R1 ES cells were cultured on irradiated mouse embryonic fibroblasts in DMEM/15% fetal bovine serum media containing LIF (ESGRO, EMD Millipore, Billerica, MA, USA) at 37°C with 5% CO_2_. For chromatin immunoprecipitation (ChIP) experiments, ES cells were cultured on gelatin-coated dishes in ES cell media containing 1.5 μM CHIR9901 (GSK3 inhibitor) for several passages to remove feeder cells. ES cells were passed by washing with phosphate-buffered saline, and dissociating with trypsin. For LSD1 inhibition experiments, ES cells were cultured in the presence of 2 μM TCP/parnate (LSD1i) for 48 h. For differentiation experiments in the absence of OCT4, ZHBTc4 ES cells [47] were infected with shLuc or shKdm5b lentiviral particles and selected in the presence of 2 μg/ml puromycin. shLuc and shKdm5b ZHBTc4 ES cells were cultured in the presence of 2 μg/ml doxycycline for 48 h to downregulate OCT4 expression. For differentiation experiements in the absence of LIF, ES cells were cultured on gelatin coated dishes or low-attachment binding dishes to promote three-dimensional formation in ES cell medium without LIF.

### Lentiviral infection

Short hairpin RNAs (shRNAs) were cloned into the pGreenPuro Vector (System Biosciences, Mountain View, CA, USA) according to the manufacture’s protocol. To generate lentiviral particles, 293 T cells were co-transfected with an envelope plasmid (plpVSVG), packaging vector (psPAX2), and shRNA expression vector using lipofectamine 2000. The medium containing lentiviral particles was harvested 24 to 48 h post-transfection and used to transduce ES cells. ES cells at 24 h post-transduction were stably selected in the presence of 1 to 2 μg/ml puromycin.

### ChIP-Seq analysis

ChIP-Seq experiments were performed as previously described with minor modifications [32]. The polyclonal KDM5B (ab50958), monoclonal H3K4me2 (ab32356), and polyclonal H3K4me1 (ab8895) antibodies were obtained from Abcam (Cambridge, MA, USA). The monoclonal H3K4me3 antibody (CS200580) was obtained from EMD Millipore. Briefly, 10^7^ to 10^8^ mouse ES cells (R1 or ZHBTc4) were harvested and chemically crosslinked with 1% formaldehyde (Sigma-Aldrich, St. Louis, MO, USA) for 5 to 10 minutes at 37°C and subsequently sonicated. Sonicated cell extracts equivalent to 2?×?10^6^ cells were used for ChIP assays. ChIP-enriched DNA was end-repaired using the End-It DNA End-Repair kit (Epicentre, Charlotte, NC, USA), followed by addition of a single A nucleotide, and ligation of PE adapters (Illumina, San Diego, CA, USA) or custom indexed adapters. PCR was performed using Phusion High Fidelity PCR master mix. ChIP libraries were sequenced on Illumina GAIIX or HiSeq platforms according to the manufacture’s protocol.

Sequence reads were mapped to the mouse genome (mm8) by bowtie [56] with settings eliminating reads mapped to multiple genomic sites. ChIP-Seq read enriched regions were identified by SICER [57] with a window size setting of 200 bp, a gap setting of 400 bp and a FDR setting of 0.001. For TFs, the ChIP-Seq read-enriched peaks were called by MACS [58] with a *P*-value setting of 0.00001.

### RNA-Seq analysis

Poly-A mRNA was purified using a Dynabeads mRNA purification kit (Life Technologies, Grand Island, NY, USA). Double-stranded cDNA was generated using a Super-Script double-stranded cDNA synthesis kit (Life Technologies). cDNA was subjected to library preparation as described above. RNA-Seq libraries were sequenced on an Illumina GAIIX or HiSeq platform according to the manufacturer’s protocol.

The RPKM measure (reads per kilobases of exon model per million reads) [59] was used to quantify the mRNA expression level of a gene from RNA-Seq data. Differentially expressed genes were identified using edgeR (FDR <0.001; fold change >1.5) [60].

### Accession number

The sequencing data from this study have been submitted to the NCBI Gene Expression Omnibus (GEO) [61] under accession number GSE53093 [62].

## Abbreviations

bp: base pair; ChIP: chromatin immunoprecipitation; ES: embryonic stem; FDR: false discovery rate; H3K4: lysine 4 of histone H3; LSD1i: LSD1 inhibitor tranylcypromine; RNAPII: RNA polymerase II; RPKM: reads per kilobases of exon model per million reads; shRNA: small hairpin RNA; SI: spreading index; TCP: tranylcypromine; TEF: transcriptional end site; TF: transcription factor; TSS: transcriptional start site.

## Competing interests

The authors declare that they have no competing interests.

## Authors’ contributions

BK conceived of the study, designed and carried out the experiments, analyzed the sequencing data, and drafted the manuscript. GH helped to analyze the sequencing data and draft the manuscript. KZ participated in the design of the study and helped to draft the manuscript. All authors have read and approved the final version of this manuscript.

## Supplementary Material

Additional file 1: Figure S1 Western blot of H3K4me1 in shLuc and shKdm5b ES cells. shLuc and shKdm5b ES cells were cultured without feeders in the presence of LIF, lysed, and subjected to western blotting for H3K4me1 using standard protocols. Click here for file
